# Seven deadly sins in artificial intelligence for digital medicine

**DOI:** 10.1038/s41746-026-02607-4

**Published:** 2026-04-15

**Authors:** Heimo Müller, Vimla L. Patel, Edward H. Shortliffe, Igor Jurisica, Andreas Holzinger

**Affiliations:** 1 Machine Learning and Information Science Group, Diagnostic and Research Center for Molecular BioMedicine, Medical University of Graz, Graz, Austria; 2 Human Machine Mind Corporation, Graz, Austria; 3 The New York Academy of Medicine, New York, NY USA; 4 Department of Biomedical Informatics, Vagelos College of Physicians and Surgeons, Columbia University, New York, NY USA; 5 Schroeder Arthritis Institute and Krembil Research Institute, University Health Network; Departments of Medical Biophysics and Computer Science, and Faculty of Dentistry, University of Toronto; Institute of Neuroimmunology, Slovak Academy of Sciences, Bratislava, Slovakia; School of Digital Public Health, Mohamed bin Zayed University of Artificial Intelligence, Abu Dhabi, UAE, Toronto, ON Canada; 6 Human-Centered AI Lab, FTEC, Department for Ecosystem Management, Climate and Biodiversity, University of Natural Resources and Life Sciences (BOKU), Vienna, Austria

**Keywords:** Business and industry, Complex networks, Complex networks, Computational biology and bioinformatics, Health care, Mathematics and computing, Psychology, Psychology, Scientific community, Social sciences

## Abstract

Artificial intelligence (AI) is increasingly embedded in clinical environments, raising questions of trust, fairness, empathy, and governance. The ethical terrain surrounding AI in medicine remains unstable despite its rapid adoption. We introduce the “Seven Deadly Sins of AI in Medicine”, a conceptual framework of recurring systemic failure modes: (i) Blind Trust, (ii) Overregulation, (iii) Dehumanization, (iv) Misaligned Optimization, (v) Overinforming and False Forecasting, (vi) Misapplied Statistics, and (vii) Self-Referential Evaluation. The framework was developed through systematic synthesis of scientific literature, clinical guidelines, and regulatory frameworks prior to any empirical data collection. To validate this pre-established framework, we conducted a global, cross-professional opinion poll of 914 stakeholders from 143 countries between July 2024 and March 2025. Results confirmed broad agreement with each pre-identified risk, revealing cross-cultural convergence in ethical concern alongside persistent divides in attitudes toward regulation—particularly between technologically advanced nations and emerging economies. We further propose an inversion of the framework into seven cardinal virtues for AI in medicine, offering actionable principles to guide responsible development and governance. The goal is to move beyond scattered ethical guidelines toward a unified diagnostic tool for trustworthy, human-centered medical AI.

## Introduction

Artificial intelligence is rapidly transforming clinical practice, from diagnostic imaging and drug discovery to administrative triage and patient monitoring^1^, to predictive modeling^2^. The promise of precision, speed, and scalability has fuelled its adoption across continents and disciplines. Yet, as AI becomes increasingly invisible and infrastructural, it also amplifies long-standing ethical and epistemic vulnerabilities in healthcare. These include opacity of decision-making, amplification of bias, overreliance on automated output, and the subtle erosion of human empathy.

Several leading bodies have articulated principles for trustworthy AI in healthcare. The European Union’s High-Level Expert Group on AI (2019) identified transparency, accountability, fairness, and human oversight as foundational requirements. The World Health Organization (2021) published guidance emphasizing safety, inclusivity, and explicability. The IEEE Global Initiative on Ethics of Autonomous and Intelligent Systems (2022) has developed standards for well-being and value alignment. Binding regulatory instruments—including the EU AI Act and the Medical Device Regulation (MDR/IVDR)—establish risk categories for high-stakes AI applications in clinical settings^3^. Collectively, these frameworks have catalyzed a global conversation about AI ethics in medicine^4–6^. Yet a persistent gap separates normative aspiration from operational reality. Clinicians, developers, and patients are left navigating uncertainty about what trustworthy AI truly entails in practice. Broad principles such as transparency or fairness, while necessary, do not by themselves translate into concrete failure-avoidance strategies. What is missing is a diagnostic vocabulary—a set of identifiable, recurring failure patterns that practitioners and governance bodies can recognize and act upon.

To address this gap, we developed the “Seven Deadly Sins of AI in Medicine” framework through systematic synthesis of three complementary source categories conducted *prior* to any survey data collection: (1) an extensive review of empirical studies, systematic reviews, and position papers identifying recurrent failure modes and systemic risks in medical AI implementation; (2) analysis of recommendations and cautionary frameworks from leading organizations, including WHO guidance on AI in healthcare, FDA guidance on AI/ML-based medical devices, professional society positions (European Society of Radiology, American Medical Association), and binding regulatory instruments (EU AI Act, MDR/IVDR, GDPR); and (3) critical synthesis and discussion by the author team, spanning medical informatics, clinical medicine, bioethics, and machine learning. The seven sins emerged as consensus failure patterns appearing consistently across all three source categories, representing systemically dangerous practices—not isolated technical errors—that manifest at the intersection of human values, clinical realities, and technological architectures. Other candidate failure modes were considered; those functioning primarily as sub-mechanisms of the seven selected categories (e.g., lack of transparency as an enabler of blind trust, or inadequate training as a contributor to dehumanization) were grouped accordingly to preserve diagnostic clarity. The deliberate use of “deadly sins” as a metaphorical frame was a rhetorical device intended to communicate the severity of these failure patterns and to engage a broad professional audience—not a theological or strictly scientific claim.

Each sin represents a distinct systemic failure mode. **Sin 1: Blind Trust ("See No Evil, Hear No Evil”)** captures the dangers of overreliance on AI systems without proper validation, context-awareness, or clinical oversight^7^. The allure of automated authority can foster uncritical acceptance even when results are opaque or non-reproducible. In high-stakes environments like oncology or emergency care, this can result in misdiagnosis, over-treatment, or delays in critical decision-making. **Sin 2: Overregulation ("No Guts, No Glory”)** recognizes that excessive or misdirected regulation can be just as harmful as under-regulation. Overly cautious frameworks may hinder innovation, restrict clinician autonomy, or delay potentially life-saving tools, particularly when regulation fails to differentiate between high-risk diagnostic AI and low-risk administrative tools^8^. **Sin 3: Dehumanization** refers to the erosion of the relational and empathetic aspects of care when AI systems are poorly integrated^9^. Automated triage bots, scripted diagnostic interfaces, or emotionless decision-support tools risk reducing patients to data points and clinicians to passive intermediaries^10^, with particular relevance for shared decision-making contexts where the doctor-patient relationship may shift from a dyad to a triad involving AI^11,12^. **Sin 4: Misaligned Optimization** reflects the tension between AI’s tendency to optimize for single objectives and medicine’s requirement for simultaneous optimization across technical, economical, and moral dimensions^13^. When proxies for success are poorly chosen, systems can perform exactly as designed and still fail their users^14^. **Sin 5: Overinforming and False Forecasting** arises when the promise of rich, real-time AI insights becomes a burden, overwhelming clinicians with excessive data, irrelevant alerts, or speculative forecasts^15^, and creating a false sense of precision where clinical uncertainty remains irreducible^16^. **Sin 6: Misapplied Statistics** occurs when population-level statistical predictions are applied indiscriminately to individual cases without contextual adjustment, producing mechanical generalization and clinical misjudgment^17,18^, a risk amplified when systems are deployed across diverse populations without accounting for demographic variability or multimorbid complexity^19^. **Sin 7: Self-Referential Evaluation** describes the practice of assessing AI systems solely on internal metrics or simulations, without external auditing, human feedback, or real-world testing—a self-referential loop that can mask bias, error propagation, and unexpected failure modes^20,21^. Due to patient and disease drifts, this cannot be a one-time validation; systematic and perpetual adjustment is required.

The seven sins framework is intended not only as a diagnostic instrument but as a springboard for positive action. Each sin implies a corresponding cardinal virtue for AI in medicine—actionable principles that, when cultivated, promote safe and trustworthy outcomes: (1) Critical validation; (2) Appropriate, proportionate regulation; (3) Human-centered design supporting shared decision-making; (4) Holistic, multi-objective optimization; (5) Transparent communication and explainable models; (6) Statistical rigor and individualization; and (7) Independent, perpetual evaluation. This positive framing is our primary message: responsible AI in medicine is the active cultivation of these virtues by clinicians, developers, and policymakers.

## Results

**Fig. 1:**
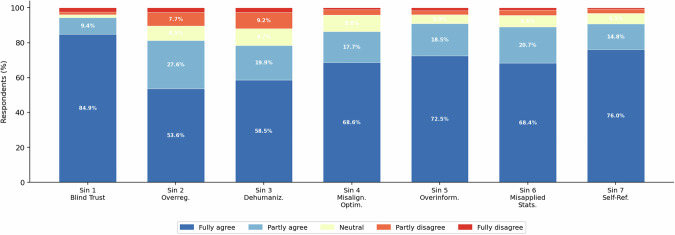
Overall agreement with the “Seven Deadly Sins of AI in Medicine” (*n* = 914). Stacked bars show the proportion of respondents in each response category (Fully agree, Partly agree, Neutral, Partly disagree, Fully disagree) for each sin: (1) Blind Trust in AI, (2) Overregulation, (3) Robotizing and Dehumanization, (4) Wrong Targets in Optimization, (5) Over-informing and False Forecasting, (6) Application of Statistical Statements to Individual Cases, and (7) Self-referential AI-based Monitoring. These results reflect agreement with pre-defined statements rather than spontaneous identification of concerns. Complete dataset: https://github.com/human-centered-ai-lab/7-sins-of-medical-ai/tree/main/RESULTS.

**Fig. 2:**
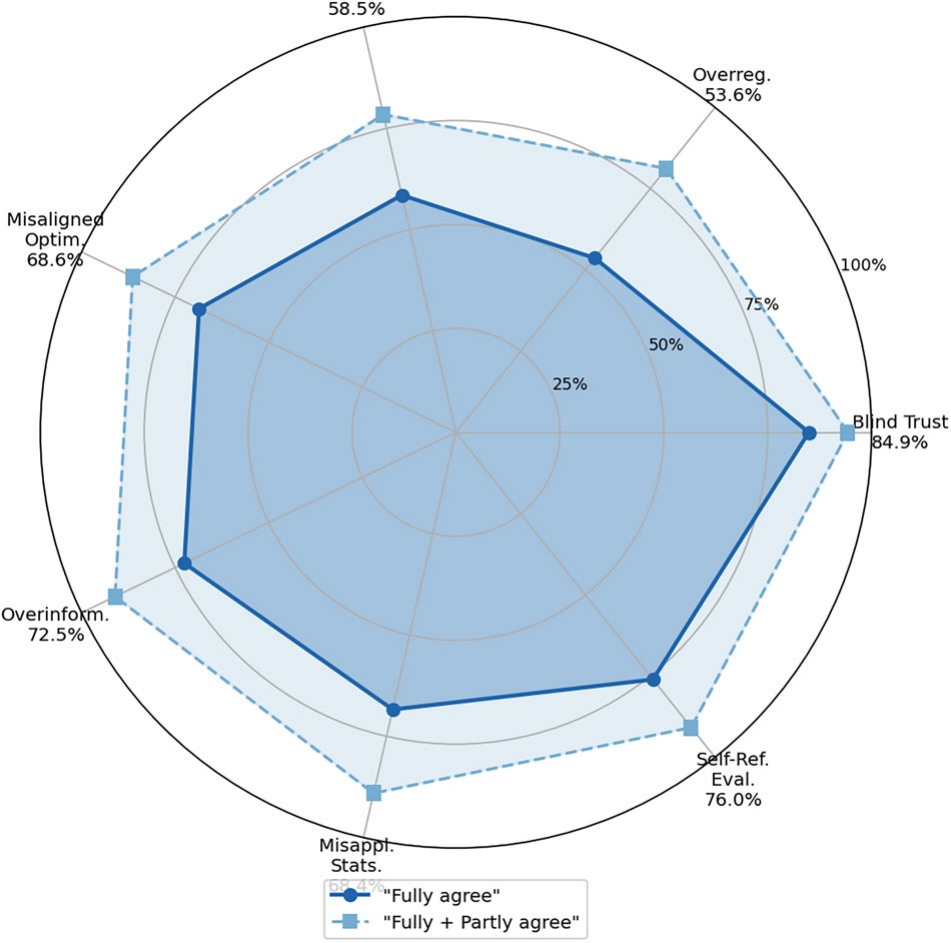
Radar chart of the stakeholder agreement profile across all seven sins. Blue filled area: proportion of respondents giving “Fully agree''; light blue outline: proportion giving “Fully agree” or “Partly agree” combined. The asymmetric profile highlights Sin 1 (Blind Trust, 84.9%) as the strongest concern, and Sin 2 (Overregulation, 53.6%) as the most contested.

The survey was completed by 915 respondents (of 940 collected; 25 excluded during preprocessing; see Methods). Participants represented diverse professional backgrounds and countries spanning both highly developed AI regions and emerging economies. Figure 1 provides an overview of response distributions across all seven sins, and Fig. 2 displays the overall agreement profile in a radar visualization (see also Supplementary Fig. 1 for the complete stacked response distribution). Before reporting individual findings, it is important to note that participants were presented with seven pre-defined statements and asked to rate agreement. The results therefore reflect *validation* of a pre-established framework rather than inductive discovery. Open comment fields were also included; qualitative analysis of these unprompted responses is presented in Supplementary Note 1.

### Sin 1: Blind Trust in AI

Blind trust was the most widely agreed-upon concern. A total of 84.9% of respondents fully agreed that over-reliance on AI without sufficient validation can result in incorrect or inappropriate decision-making, potentially causing harm. An additional 9.4% partly agreed. Only a small minority expressed disagreement (2.3% fully disagree, 1.5% partly disagree) or neutrality (1.9%). Striking outliers were the youngest age group (<20 years, 68.6% full agreement) and respondents in public authority roles (68.6%), both substantially below the overall rate (Supplementary Fig. 2). Older respondents showed higher concern: 88.8% of the 40–59 group and 88.0% of those above 60 fully agreed. Among professions, medicine showed the highest full agreement at 90.6%.

### Sin 2: Over-Regulation

Opinions on regulation were more divided than for any other sin. Approximately 53.6% fully agreed, and 27.6% partly agreed, that excessive regulation could hinder innovation and experimentation. A minority disagreed (2.6% fully, 7.7% partly), while 8.5% remained neutral. Geographical differences were notable: respondents from AI-mature economies showed higher concern about overregulation (see Fig. 4) than those from emerging economies. The youngest respondents showed the lowest full agreement (40.0% for <20 years; 42.3% for 20–39 years), while those above 40 agreed substantially more (63.3% for 40–59; 66.0% for >60). Among professions, medicine (68.6%) and management (67.1%) showed the strongest concern.

### Sin 3: Robotizing and Dehumanization

Around 58.5% of respondents fully agreed and 19.9% partly agreed that AI integration may lead to a loss of empathy and lower patient satisfaction. A small minority disagreed (2.6% fully, 9.2% partly), while 9.7% were neutral. Notably, the medical profession showed strong concern at 73.7% full agreement, as did respondents under 20 years (71.4%). Public authority showed the lowest full agreement (54.3%).

### Sin 4: Wrong Targets in Optimization

A large majority (68.6% fully agree, 17.7% partly agree) expressed concern that AI systems optimize for metrics not aligned with meaningful human outcomes. Only a small share disagreed (1.0% fully, 3.1% partly) or remained neutral (9.6%). Strongest agreement was in management (81.4%) and the medical profession (81.3%). The youngest age group showed notably lower concern (62.9% full agreement).

### Sin 5: Over-Informing and False Forecasting

Roughly 72.5% fully agreed and 18.5% partly agreed that excessive or misleading AI-generated information can reduce trust and cause confusion. Disagreement was minimal (1.5% fully, 2.4% partly), and only 5.0% remained neutral. Medicine showed the highest professional agreement (84.3%), while public authority agreed less (62.9%). The 40–59 age group showed higher concern (78.2%) than younger respondents.

### Sin 6: Application of Statistical Statements to Individual Cases

About 68.4% fully agreed, and 20.7% partly agreed, that misuse of statistical models for individual decision-making can lead to inappropriate outcomes. Few disagreed (1.3% fully, 3.1% partly). Public authority showed only 57.1% full agreement, and the youngest age group likewise showed lower concern. Medicine (80.4%) and management (79.5%) showed the highest professional agreement.

### Sin 7: Self-Referential AI-Based Monitoring

About 76.0% fully agreed and 14.8% partly agreed that AI systems evaluating themselves without external oversight risk reduced accountability and transparency. Disagreement was negligible (0.7% fully, 2.4% partly), with 6.1% neutral. Public authority again showed the lowest full agreement (65.7%), while medicine (84.0%) and management (81.4%) showed the highest.

### Qualitative Analysis of Open Comments

Each survey item included an optional open comment field, and participants could also leave a general comment at the end. Across all items, 1,004 non-empty comment entries were submitted (approximately 1.1 entries per respondent on average), providing an important complement to the Likert-scale data. A thematic analysis of these unprompted responses broadly confirmed the pre-defined framework while also surfacing several cross-cutting themes.

For Sin 1 (Blind Trust), the dominant unprompted theme was the necessity of rigorous clinical validation (raised in 23% of relevant comments), with several respondents emphasizing the risk of progressive de-skilling—that habitual reliance on AI may erode the clinical reasoning capacity needed to catch AI errors. For Sin 2 (Overregulation), a quarter of commenters spontaneously addressed concerns about excessive restriction of innovation, mirroring the Likert-scale division. For Sin 3 (Dehumanization), empathy and the patient relationship were the most frequently raised themes (35% of comments), with a recurring argument that AI should function as a tool supporting clinicians, not as a replacement for human interaction. For Sin 7 (Self-Referential Evaluation), three themes emerged with equal frequency: demand for human oversight, the need for external validation, and transparency and accountability requirements (each cited in approximately 10% of comments).

Notably, several general comments from respondents in sub-Saharan Africa and other under-served regions emphasized that AI represents a critical opportunity to address severe shortages of clinical personnel—a perspective rarely voiced in the seven sins framing. One respondent wrote that their community “ha[d] no doctors,” framing AI not as a risk but as an urgent necessity. This finding underscores the importance of geographically sensitive AI governance. A small number of commenters also critiqued the survey instrument itself—noting that the question framing was “leading” toward agreement, reinforcing the epistemic limitation already acknowledged above. Full verbatim comments are available in the public dataset and a structured thematic analysis is presented in Supplementary Note 1.

### Cross-Group Comparisons

**Fig. 3:**
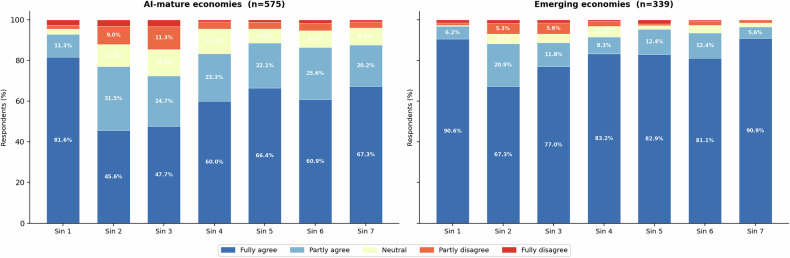
Full response distributions by country category. AI-mature economies (*n* = 575, left panel) and emerging economies (*n* = 339, right panel). AI-mature economies show consistently higher full agreement across most sins, most prominently for Sin 2 (Overregulation) and Sin 3 (Dehumanization).

**Fig. 4:**
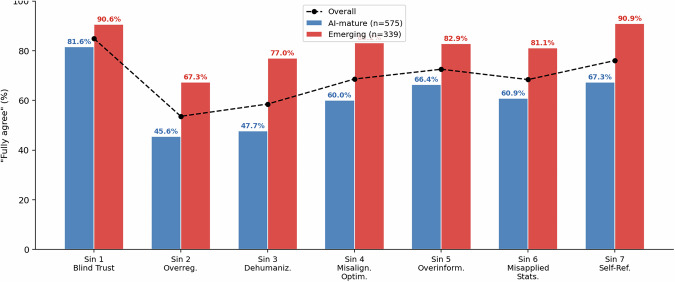
"Fully agree” rates by country category (AI-mature vs. emerging economies) for each of the seven sins. The dashed black line represents the overall sample average. AI-mature economies consistently report higher concern, particularly for Sins 2, 3, and 5. Emerging economies show notably lower concern for overregulation.

**Fig. 5:**
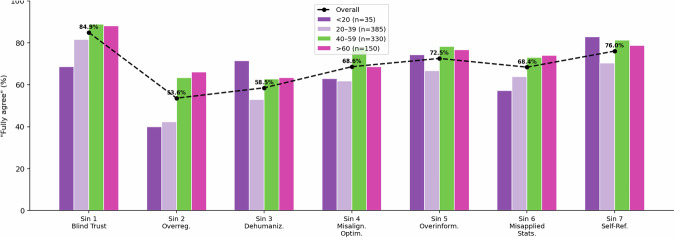
"Fully agree” rates by age group for each of the seven sins. The dashed black line shows the overall sample average with exact percentage labels. Respondents under 20 years consistently show the lowest concern across most sins, while those aged 40--59 and above 60 show the highest. The gap is most pronounced for Sins 1 (Blind Trust) and 2 (Overregulation).

**Fig. 6:**
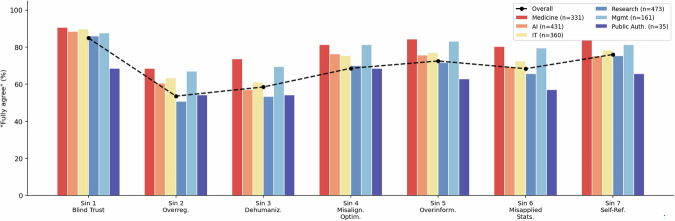
"Fully agree” rates by professional background for each of the seven sins. The dashed black line shows the overall average. Medicine and management consistently show the highest concern; public authority shows the lowest agreement across all sins. The profession-level variation is most notable for Sin 2 (Overregulation) and Sin 3 (Dehumanization).

Figures 3–4 present response distributions by country category, Figs. 5–6 present breakdowns by age group and professional background.

### Summary

Epistemic risks commanded the highest agreement: blind trust (84.9%), self-referential evaluation (76.0%), and false forecasting (72.5%) ranked highest. Overregulation elicited the most divided responses (53.6% full agreement), reflecting global disagreement about the appropriate level of AI governance. A consistent pattern across all subgroups was that public authority respondents and the youngest age group (<20 years) showed systematically lower agreement across nearly all sins, while respondents in medicine, management, and those aged 40 and over showed the strongest concern.

## Discussion

The survey results confirm that the seven pre-defined failure patterns are regarded as genuine risks by a broad and professionally diverse global sample. It is essential to interpret these findings precisely: because participants were presented with a pre-defined list of concerns, the study demonstrates *stakeholder validation* of the Seven Deadly Sins framework, not that participants would have independently converged on the same seven categories without prompting. This is a more limited—but still valuable—finding than a claim of independent consensus. To complement this, open comment fields were included, and qualitative analysis of these unprompted responses is available in Supplementary Note 1. Of the seven concerns, overregulation stands out as the least universally recognized risk, with only 53.6% fully agreeing compared with 84.9% for blind trust. This signals prevailing ambivalence: some see regulation as a barrier to innovation, while others view the absence of governance as the greater threat. By contrast, epistemic concerns—blind trust, self-referential evaluation, and false forecasting—command near-universal agreement, suggesting that the gradual erosion of human judgment through feedback loops and automation bias is the most widely shared concern. Concerns about dehumanization showed moderate agreement (58.5% full agreement), marking a growing recognition that AI systems risk eroding the relational fabric of care. In high-stakes domains where empathy, trust, and discretion are central—not peripheral—this is especially troubling. AI increasingly mediates doctor-patient dynamics and shared decision-making^11,12^, and the data reflect genuine concern about this shift across professional groups.

Our survey revealed substantial consistency across countries in identifying the same ethical concerns as important, particularly for epistemic risks. However, notable divergences emerged, especially for overregulation (Sin 2) and dehumanization (Sin 3). Respondents from AI-mature economies showed meaningfully higher concern about overregulation than those from emerging economies, consistent with the interpretation that experience of real-world AI deployment—including its failures—heightens demand for governance. This consistency defies common assumptions that technology attitudes differ sharply across cultural and economic divides, while the divergences highlight where context-sensitive regulation is most needed. This emerging cross-cultural ethical vocabulary represents an opportunity to develop globally harmonized frameworks that move beyond technical compliance toward shared values^22^. The survey deliberately excluded politicians and business owners, to ensure that political and commercial agendas did not influence data interpretation.

Countries with the most advanced AI infrastructures were also the most vocal in demanding tighter regulation. The answer lies in experience: in technologically mature healthcare systems, the risks of AI are no longer theoretical. These countries have witnessed algorithmic misdiagnoses, equity gaps, explainability failures, and clinician burnout from over-automated workflows. Regulation, for them, is not a barrier to innovation but a mechanism of trust restoration. By contrast, countries still in earlier phases of digital transformation often frame AI as a strategic opportunity and regard regulation as a risk to progress. This divergence poses a significant challenge for global AI governance: if regulation is fragmented along developmental lines, we risk a two-speed world in which affluent countries impose strict ethical constraints while emerging economies adopt AI under looser oversight, potentially importing systems not calibrated to their populations. Harmonized global standards—such as those explored by WHO, OECD, and the EU AI Act—must remain sensitive to national contexts while upholding core principles of transparency, fairness, and accountability.

A consistent and noteworthy finding was that respondents working in public authority showed the lowest full agreement across all seven sins. This is particularly significant because policymakers and regulators are among the key actors responsible for shaping the governance landscape of AI in healthcare. The gap between their perceived risk levels and those of clinicians, AI researchers, and management professionals suggests that current regulatory discussions may be insufficiently informed by the concerns that practitioners and domain experts have already identified as critical.

Respondents under 20 years consistently showed the lowest concern across most sins, particularly for blind trust (68.6% vs. 84.9% overall) and overregulation (40.0% vs. 53.6% overall). This generation has grown up with AI-mediated tools and may have a different—potentially less critical—baseline attitude toward algorithmic authority. Conversely, those aged 40 and above showed the highest concern, consistent with clinical and professional experience of AI’s limitations. These findings point to the need for AI literacy education that fosters critical engagement with algorithmic systems from an early age.

The findings have several practical implications. AI-mediated interactions may alter triage, intake, and disclosure dynamics^23–25^; clinicians will need new competencies for co-navigating patient trust in AI, managing misperceptions, and intervening when machine outputs conflict with clinical judgment^26–28^. Developers must move beyond performance optimization to incorporate relational design principles—tone, timing, feedback—that influence user acceptance^29–31^. Trust in AI cannot be mandated through policy alone; it must be earned through interpretability, reproducibility, and contextual fidelity^32,33^. The risk of delegating empathy to machines remains a central ethical concern: simulated compassion can blur the line between support and manipulation, and empathy fatigue among clinicians could be exacerbated if AI becomes an emotional substitute rather than a partner^34–36^.

Several limitations should be acknowledged. First, presenting participants with pre-defined statements measures agreement rather than eliciting unprompted priorities; findings reflect validation of our framework, not independent discovery. Second, recruitment through professional networks introduces selection bias toward individuals already engaged with healthcare technology communities. Third, the exclusion of politicians and business owners, though intentional, limits stakeholder coverage, and future work with adequate funding should include these groups. Fourth, the dichotomization of countries into “AI-mature” and “emerging” categories simplifies a continuous spectrum. Fifth, a single time-point survey (July 2024–March 2025) may not capture rapidly evolving attitudes in a fast-moving regulatory landscape, particularly given concurrent EU AI Act implementation.

The Seven Deadly Sins of AI in Medicine provide both a diagnostic framework for recognizing systemic failure modes and—through their inversion into seven cardinal virtues—a positive vision for trustworthy, human-centered AI in clinical practice. Our global stakeholder survey of 914 participants from 143 countries confirmed that these concerns are broadly recognized across professional backgrounds and age groups. Epistemic risks commanded the highest agreement; overregulation elicited the most culturally divided responses. A consistent cross-group finding was the lower concern among the youngest respondents and those in public authority—the latter pointing to an important governance gap that must be addressed. Moving from the seven sins toward the seven virtues—critical validation, proportionate regulation, human-centered design, holistic optimization, transparent communication, statistical rigor, and independent evaluation—represents the path from scattered ethical principles to operational trustworthiness in medical AI^37,38^.

## Methods

### Framework Development

The Seven Deadly Sins framework was developed *prior* to the empirical survey through systematic synthesis of: (a) peer-reviewed scientific literature on AI failure modes and ethical risks in healthcare; (b) guidelines and policy documents from leading regulatory bodies, including the EU High-Level Expert Group on AI (2019), WHO (2021), FDA guidance on AI/ML-based medical devices, and the EU AI Act; and (c) critical deliberation by the author team. The seven categories represent recurrent, consensus failure patterns identified across all three source types.

### Survey Design

The survey comprised two components: (1) demographic items covering age group, professional background, country of residence; and (2) seven Likert-scale statements, each operationalizing one of the seven identified failure patterns. Respondents rated agreement on a five-point scale from “Fully Agree” to “Fully Disagree.” An open comment field was included at the end for unprompted feedback. The survey was developed and administered using EUSurvey (https://ec.europa.eu/eusurvey), the European Commission’s official survey tool, which is open-source (EUPL), GDPR-compliant, and supports anonymous response collection. The survey landing page is: https://human-centered.ai/7-sins-of-medical-ai. The survey was open from 17 July 2024 to 18 March 2025 (244 days).

### Country Categorization

For subgroup analyses, respondents were grouped into two categories based on their country of residence. **AI-mature economies** were defined as countries with established national AI strategies, significant investment in AI research and healthcare digitalization, and active AI governance frameworks as of 2024. This group includes OECD member states and other nations with comparably advanced AI infrastructure: USA, Germany, Austria, Australia, Canada, UK, France, Switzerland, Belgium, Japan, Netherlands, Sweden, Denmark, Norway, Finland, Italy, Spain, Portugal, Czech Republic, Hungary, Romania, Poland, Greece, Ireland, New Zealand, South Korea, and Singapore. All remaining countries were classified as **emerging economies**. We acknowledge that this binary classification simplifies a continuous spectrum of AI readiness and governance maturity; a more granular index-based classification is discussed as a limitation above.

### Participants and Recruitment

Participants were recruited through direct email outreach via professional networks, announcements at international conferences in medical informatics, AI, and bioethics, and promotion through professional association mailing lists and online communities. No platform registration was required. Politicians and business owners were intentionally excluded to minimize the influence of political and commercial agendas on responses; this decision was also constrained by the available study budget. The survey was accessible to anyone with the invitation link; no financial incentives were provided. Inclusion criteria were: active or recent professionals in medicine, nursing, computer science, AI/machine learning, bioethics, or healthcare management, or individuals with relevant patient experience of AI-supported care. Exclusion criteria were: incomplete responses (fewer than 50% of items); duplicate entries; and individuals identifying exclusively as politicians or business owners with no healthcare or technology background.

### Participant Demographics

A total of 940 responses were collected; 25 were excluded during preprocessing, yielding an analytic sample of *n* = 914 (note: one additional response was excluded between preliminary and final preprocessing, yielding 914 rather than 915 in the final analytic dataset). Detailed participant characteristics are presented in Table 1.

**Table 1:** Participant demographic characteristics (*n* = 914)

Characteristic	Category	*n* (%)
Age group	Below 20 years	35 (3.8%)
	20–39 years	385 (42.1%)
	40–59 years	330 (36.1%)
	Above 60 years	150 (16.4%)
Professional field^a^	Research	473 (51.8%)
	Artificial Intelligence	431 (47.2%)
	Information Technology	360 (39.4%)
	Medicine	331 (36.2%)
	Management	161 (17.6%)
	Decision-Making	103 (11.3%)
	Public Authority	35 (3.8%)
Country category	AI-mature economies (OECD, established AI governance)	575 (62.9%)
	Emerging economies	339 (37.1%)
	Total unique countries represented	143
Top 5 countries	USA	124 (13.6%)
	Germany	84 (9.2%)
	Austria	84 (9.2%)
	India	64 (7.0%)
	Australia	30 (3.3%)

^a^Participants could select multiple professional fields; percentages sum to more than 100%. “Not reported” for age: 0 respondents.

### Statistical Analysis

Descriptive statistics were used to summarize response distributions for each Likert-scale item across the full sample and across demographic sub-groups (age, profession, country category). Proportions of respondents in each response category are reported for each sin. Sub-group comparisons are reported descriptively. Open-text comments were analyzed using qualitative thematic analysis; findings are reported in Supplementary Note 1. All data and analysis scripts are available at https://github.com/human-centered-ai-lab/7-sins-of-medical-ai.

### Ethics Approval and Consent to Participate

This study involved an anonymous, voluntary opinion survey of adult participants and did not collect personal identifiers, sensitive health data, or information enabling re-identification. The survey instrument consisted of seven Likert-scale items and three demographic variables (age group, professional background, and country category), designed to minimize data collection while ensuring analytical relevance.

In accordance with applicable European data protection regulations (GDPR) and the institutional policies of the Medical University of Graz, Austria, the study was assessed by the Ethics Committee of the Medical University of Graz (Ethikkommission der Medizinischen Universität Graz) as exempt from formal ethics committee approval. The basis for this exemption was that the study did not involve patient data, clinical interventions, vulnerable populations, biosamples, or any information enabling re-identification of participants. No application reference number was issued, as the exemption determination did not require a formal submission under applicable institutional and national regulations for minimal-risk social science research. No experimental manipulation or deception was employed.

All participants were fully informed prior to participation about the purpose of the study, the voluntary nature of their involvement, the anonymous processing of responses, and the intended open dissemination of aggregated results. Proceeding to complete the survey after reading the participant information sheet constituted informed consent. Participants were free to withdraw at any time without consequence. Data were stored in anonymized form and analysed only at the aggregate level; no IP addresses or tracking information were retained. The study was conducted in accordance with the principles of the Declaration of Helsinki and established guidelines for ethical research involving human participants in digital survey contexts.

## Supplementary information

Supplementary Information

## Data Availability

All anonymized data, all figures, and analysis scripts are openly available at (https:/github.com/human-centered-ai-lab/7-sins-of-medical-ai).

## Code availability

Code for data visualisation and statistical analysis is available in the same repository under an open-source licence.

## References

[1] Rajpurkar, P., Chen, E., Banerjee, O. & Topol, E. J. AI in health and medicine. *Nat. Med.* **28**, 31–38 (2022). 35058619 10.1038/s41591-021-01614-0

[2] Rajkomar, A. et al. Scalable and accurate deep learning with electronic health records. *npj Digital Med.* **1**, 18 (2018). 10.1038/s41746-018-0029-1 PMC6550175 31304302

[3] Shick, A. A. et al. Transparency of artificial intelligence/machine learning-enabled medical devices. *npj Digital Med.* **7**, 21 (2024). 10.1038/s41746-023-00992-8 PMC10810855 38273098

[4] Paulus, J. K. & Kent, D. M. Predictably unequal: understanding and addressing concerns that algorithmic clinical prediction may increase health disparities. *npj Digital Med.* **3**, 99 (2020). 10.1038/s41746-020-0304-9 PMC7393367 32821854

[5] Comeau, D. S., Bitterman, D. S. & Celi, L. A. Preventing unrestricted and unmonitored AI experimentation in healthcare through transparency and accountability. *npj Digital Med.* **8**, 42 (2025). 10.1038/s41746-025-01443-2 PMC11743128 39827300

[6] Holzinger, A., Zatloukal, K. & Müller, H. Is human oversight to AI systems still possible?. *N. Biotechnol.* **85**, 59–62 (2025). 39675423 10.1016/j.nbt.2024.12.003

[7] Klingbeil, A., Grützner, C. & Schreck, P. Trust and reliance on AI: An experimental study on the extent and costs of overreliance on AI. *Computers Hum. Behav.* **160**, 108352 (2024).

[8] Saenz, A. D., Harned, Z., Banerjee, O., Abràmoff, M. D. & Rajpurkar, P. Autonomous AI systems in the face of liability, regulations and costs. *npj Digital Med.* **6**, 185 (2023). 10.1038/s41746-023-00929-1 PMC10558567 37803209

[9] Akingbola, A., Adeleke, O., Idris, A., Adewole, O. & Adegbesan, A. Artificial intelligence and the dehumanization of patient care. *J. Med. Surg. Public Health* **3**, 100138 (2024).

[10] Sharma, D. et al. Triage-Bot: An assistive triage framework. In *2024 IEEE International Conference on Digital Health (ICDH)*, 138–140 (IEEE, 2024).

[11] Caruso, I. et al. Artificial intelligence and the doctor–patient relationship expanding the paradigm of shared decision making. *Bioethics* **37**, 424–432 (2023). 36964989 10.1111/bioe.13158

[12] Sauerbrei, A., Kerasidou, A., Lucivero, F. & Hallowell, N. The impact of artificial intelligence on the person-centred, doctor–patient relationship: some problems and solutions. *BMC Med. Inform. Decis. Mak.* **23**, 73 (2023). 37081503 10.1186/s12911-023-02162-y PMC10116477

[13] Vamplew, P., Dazeley, R., Foale, C., Firmin, S. & Mummery, J. Human-aligned artificial intelligence is a multiobjective problem. *Ethics Inf. Technol.* **20**, 27–40 (2018).

[14] Yoon, Y., Guimaraes, T. & O’Neal, Q. Exploring the factors associated with expert systems success. *MIS Q.* **19**, 83–106 (1995).

[15] Mahajan, A. & Gilbert, S. Do we need AI guardians to protect us from health information overload?. *npj Digital Med.* **8**, 632 (2025). 10.1038/s41746-025-02093-0 PMC12559249 41145602

[16] Zhou, J., Müller, H., Holzinger, A. & Chen, F. Ethical ChatGPT: Concerns, challenges, and commandments. *Electronics* **13**, 3417 (2024).

[17] Prosperi, M. et al. Causal inference and counterfactual prediction in machine learning for actionable healthcare. *Nat. Mach. Intell.* **2**, 369–375 (2020).

[18] Thiese, M. S., Arnold, Z. C. & Walker, S. D. The misuse and abuse of statistics in biomedical research. *Biochemia Med.* **25**, 5–11 (2015). 10.11613/BM.2015.001 PMC4401313 25672462

[19] Majnarić, L. T., Babič, F., O’Sullivan, S. & Holzinger, A. AI and big data in healthcare: Towards a more comprehensive research framework for multimorbidity. *J. Clin. Med.* **10**, 766 (2021). 33672914 10.3390/jcm10040766 PMC7918668

[20] Mahajan, S. The executioner paradox: understanding self-referential dilemma in computational systems. *AI Soc.* **40**, 1939–1946 (2025).

[21] Mathews, S. C. et al. Digital health: a path to validation. *npj Digital Med.* **2**, 38 (2019). 10.1038/s41746-019-0111-3 PMC6550273 31304384

[22] Mueller, H., Mayrhofer, M. T., van Veen, E.-B. & Holzinger, A. The ten commandments of ethical medical AI. *IEEE Computer* **54**, 119–123 (2021).

[23] Pairon, A., Philips, H. & Verhoeven, V. A scoping review on the use and usefulness of online symptom checkers and triage systems: how to proceed?. *Front. Med.* **9**, 1040926 (2023). 10.3389/fmed.2022.1040926 PMC9853165 36687416

[24] Wallace, W. et al. The diagnostic and triage accuracy of digital and online symptom checker tools: a systematic review. *npj Digital Med.* **5**, 118 (2022). 10.1038/s41746-022-00667-w PMC9385087 35977992

[25] Pickard, M. D., Roster, C. A. & Chen, Y. Revealing sensitive information in personal interviews: Is self-disclosure easier with humans or avatars and under what conditions?. *Comput. Hum. Behav.* **65**, 23–30 (2016).

[26] Bloice, M., Simonic, K.-M. & Holzinger, A. Casebook: a virtual patient iPad application for teaching decision-making through the use of electronic health records. *BMC Med. Inform. Decis. Mak.* **14**, 1–9 (2014). 25100051 10.1186/1472-6947-14-66 PMC4149039

[27] De Togni, G., Erikainen, S., Chan, S. & Cunningham-Burley, S. What makes AI ‘intelligent’ and ‘caring’? Exploring affect and relationality across three sites of intelligence and care. *Soc. Sci. Med.* **277**, 113874 (2021). 33901725 10.1016/j.socscimed.2021.113874 PMC8135128

[28] Holzinger, A. & Mueller, H. Toward human-AI interfaces to support explainability and causability in medical AI. *IEEE Computer* **54**, 78–86 (2021).

[29] Topol, E. J. High-performance medicine: the convergence of human and artificial intelligence. *Nat. Med.* **25**, 44–56 (2019). 30617339 10.1038/s41591-018-0300-7

[30] Loveys, K., Sebaratnam, G., Sagar, M. & Broadbent, E. The effect of design features on relationship quality with embodied conversational agents: a systematic review. *Int. J. Soc. Robot.* **12**, 1293–1312 (2020).

[31] Holzinger, A. et al. Personas for artificial intelligence (AI) an open source toolbox. *IEEE Access* **10**, 23732–23747 (2022).

[32] Cabitza, F., Campagner, A. & Balsano, C. Bridging the “last mile” gap between AI implementation and operation: “data awareness” that matters. *Ann. Transl. Med.* **8**, 501 (2020). 32395545 10.21037/atm.2020.03.63 PMC7210125

[33] Zuchowski, L. C., Zuchowski, M. L. & Nagel, E. A trust based framework for the envelopment of medical AI. *npj Digital Med.* **7**, 230 (2024). 10.1038/s41746-024-01224-3 PMC11350073 39191927

[34] Arnold, M. H. Teasing out artificial intelligence in medicine: an ethical critique of artificial intelligence and machine learning in medicine. *J. Bioethical Inq.* **18**, 121–139 (2021). 10.1007/s11673-020-10080-1 PMC7790358 33415596

[35] Longoni, C., Bonezzi, A. & Morewedge, C. K. Resistance to medical artificial intelligence. *J. Consum. Res.* **46**, 629–650 (2019).

[36] Nag, P. K., Bhagat, A. & Priya, R. V. Expanding AI’s role in healthcare applications: a systematic review of emotional and cognitive analysis techniques. *IEEE Access* **13**, 69129–69160 (2025).

[37] Okolo, C. T. Optimizing human-centered AI for healthcare in the Global South. *Patterns* **3**, 100421 (2022). 35199066 10.1016/j.patter.2021.100421 PMC8848006

[38] Rao, V. M. et al. Multimodal generative AI for medical image interpretation. *Nature* **639**, 888–896 (2025). 40140592 10.1038/s41586-025-08675-y

