# Supplementary material for: Seven deadly sins in artificial intelligence for digital medicine
Source: NPJ Digit Med. 2026 Apr 15;9:540. doi: 10.1038/s41746-026-02607-4 (PMC13369183; doi:10.1038/s41746-026-02607-4)
Supplement: Supplementary file 1 — Supplementary Information [file 41746_2026_2607_MOESM1_ESM.pdf]

Supplementary Information

Seven Deadly Sins in Artificial Intelligence for Digital Medicine

Heimo Müller[1,2], Vimla L. Patel[3,4], Edward H. Shortliffe[4], Igor Jurisica[5], and Andreas Holzinger*[1,6]

[1]Diagnostic and Research Center for Molecular BioMedicine, Medical University of Graz, Austria

[2]Human Machine Mind Corporation, Graz, Austria

[3]The New York Academy of Medicine, New York, USA

[4]Department of Biomedical Informatics, Columbia University, New York, USA

[5]Schroeder Arthritis Institute, University Health Network; Departments of Computer Science and Medical Biophysics, University of Toronto, Canada

[6]Human-Centered AI Lab, University of Natural Resources and Life Sciences Vienna (BOKU), Austria

*Corresponding author: andreas.holzinger@human-centered.ai

Contents

Supplementary Note 1 .... p. 3

Supplementary Figure 1 .... p. 7

Supplementary Figure 2 .... p. 8

Supplementary Note 1: Thematic Analysis of Open Comments

Each of the seven survey items included an optional open-text comment field, and respondents could additionally leave a free-form comment at the end of the survey. Across all items, 1,004 non-empty comment entries were submitted (approximately 1.1 entries per respondent on average). These unprompted responses were subjected to inductive thematic analysis to identify recurring concerns, elaborations, and critiques that complement the quantitative Likert-scale data. The qualitative analysis was conducted independently of the pre-defined framework and served as a convergent validity check.

Sin 1 — Blind Trust in AI

The dominant theme in comments related to Sin 1 was the necessity of rigorous clinical validation before deployment (raised in approximately 23% of relevant comments). A notable secondary theme was progressive *de-skilling*: several respondents expressed concern that habitual reliance on AI recommendations may erode the independent clinical reasoning capacity needed to identify and correct AI errors. A smaller subset of comments emphasised the importance of transparency and explainability as prerequisites for appropriate trust calibration.

Sin 2 — Overregulation

Approximately one quarter of commenters for Sin 2 spontaneously raised concerns about excessive restriction of innovation, mirroring the Likert-scale division observed in the quantitative results. A contrasting minority of comments argued that current regulatory frameworks are insufficient and that voluntary guidelines lack enforcement mechanisms. Several respondents from emerging economies explicitly framed regulation as a barrier to equitable access to beneficial AI technologies.

Sin 3 — Dehumanization

Empathy and the patient relationship were the most frequently raised themes in comments for Sin 3, appearing in approximately 35% of relevant entries. The most common argument was that AI should function as a supportive tool for clinicians rather than as a replacement for human interaction. A recurring concern was the risk that emotionally neutral or scripted AI interfaces may inadvertently communicate a lack of care, even where the underlying system is technically proficient. Several commenters highlighted shared decision-making as a particularly vulnerable context.

Sin 4 — Misaligned Optimization

Comments for Sin 4 frequently highlighted the distinction between process metrics (e.g., throughput, diagnostic accuracy) and outcome metrics meaningful to patients (e.g., quality of life, long-term wellbeing). Several respondents with management backgrounds noted that institutional incentive structures often reward the former while neglecting the latter. A smaller set of comments raised concerns about equity: that optimization objectives trained predominantly on majority-population data may systematically underperform for minority or underserved patient groups.

Sin 5 — Overinforming and False Forecasting

Alert fatigue was the most prominent theme in comments related to Sin 5. Respondents with clinical backgrounds frequently described experiences of being overwhelmed by irrelevant or low-confidence AI-generated notifications, leading to desensitisation and reduced vigilance. A second theme concerned the communication of probabilistic predictions to patients: several commenters noted that patients often interpret statistical risk estimates as individual certainties, particularly when these are presented without appropriate contextualisation.

Sin 6 — Misapplied Statistics

Comments for Sin 6 emphasised the gap between population-level model training and individual-level clinical application. Several respondents highlighted that confidence intervals and model performance metrics are rarely communicated to end users, creating an illusion of certainty. Concerns about demographic and cultural transferability were prominent among respondents from non-Western countries, who noted that models trained predominantly on data from high-income countries may perform poorly in their clinical contexts.

Sin 7 — Self-Referential AI-Based Monitoring

Three themes emerged with approximately equal frequency (each cited in approximately 10% of comments): (i) demand for mandatory human oversight in AI evaluation processes; (ii) the need for external, independent validation using prospective real-world data; and (iii) requirements for transparency and public accountability in reporting AI performance. Several respondents explicitly called for regulatory requirements mandating post-market surveillance and continuous monitoring protocols analogous to those applied to medical devices.

Cross-Cutting Themes

Several themes appeared across multiple sin categories. First, respondents in sub-Saharan Africa and other under-served regions frequently reframed the seven sins from the perspective of AI as an urgent clinical necessity rather than a risk: where specialist medical workforce is critically scarce, the risks of *not* deploying AI may outweigh those described in the framework. This perspective was rarely voiced in comments from high-income countries and underscores the importance of geographically sensitive AI governance. Second, a small number of commenters critiqued the survey instrument itself, noting that the question framing was “leading” toward agreement—an epistemic limitation explicitly acknowledged in the main text. Third, several respondents called for greater inclusion of patient voices and lay

perspectives in AI governance processes, beyond the professional stakeholders targeted in this study.

Full verbatim comments are available in the public dataset at: https://github.com/human-centered-ai-lab/7-sins-of-medical-ai.

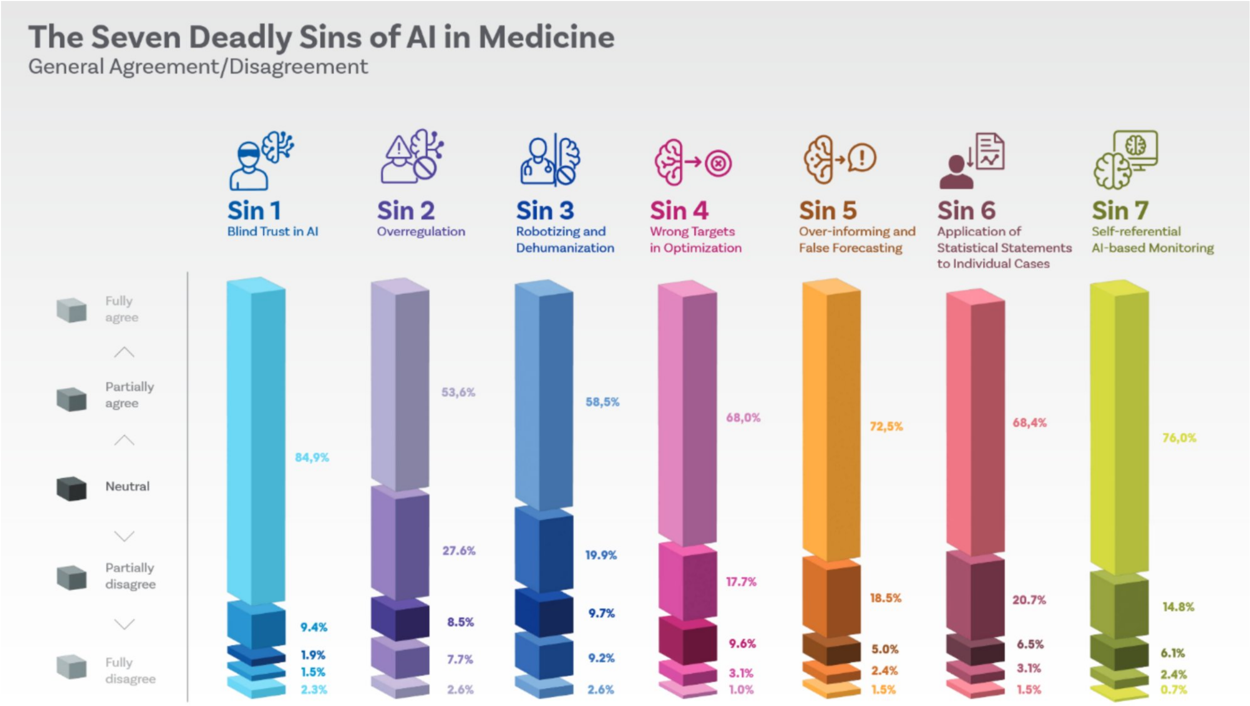

Supplementary Figure 1: Overall agreement and disagreement with the "Seven Deadly Sins of AI in Medicine" ($n = 914$). Stacked bars show the proportion of respondents in each of five response categories (Fully agree, Partly agree, Neutral, Partly disagree, Fully disagree) for each of the seven pre-defined failure modes: (1) Blind Trust in AI, (2) Overregulation, (3) Robotizing and Dehumanization, (4) Wrong Targets in Optimization, (5) Over-informing and False Forecasting, (6) Application of Statistical Statements to Individual Cases, and (7) Self-referential AI-based Monitoring. These results reflect agreement with pre-defined statements rather than spontaneous identification of concerns. Complete dataset available at: https://github.com/human-centered-ai-lab/7-sins-of-medical-ai/tree/main/RESULTS.

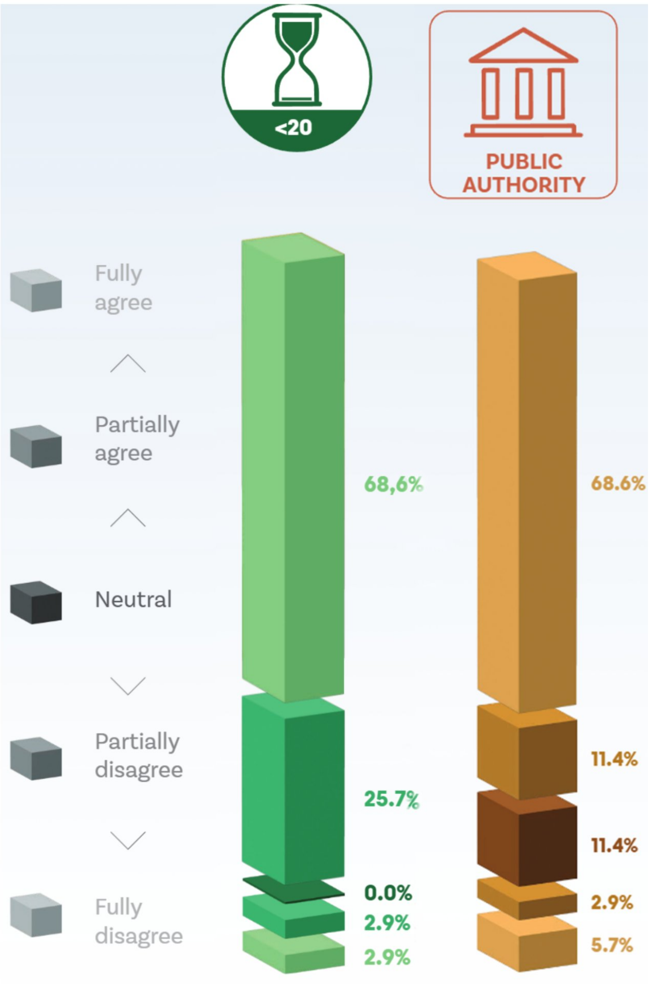

Supplementary Figure 2: Sin 1 (Blind Trust in AI) — response distributions for notable outlier subgroups. Response distributions for two subgroups showing substantially lower full agreement than the overall sample: respondents under 20 years of age (<20 years; green bars) and respondents working in public authority roles (orange bars). Both groups show 68.6% full agreement, compared with the overall sample average of 84.9%. Divergence from the overall population (dashed line) is most pronounced at the "Fully agree" category.
